# Consumer-Guided Development of an Engagement-Facilitation Intervention for Increasing Uptake and Adherence for Self-Guided Web-Based Mental Health Programs: Focus Groups and Online Evaluation Survey

**DOI:** 10.2196/22528

**Published:** 2020-10-29

**Authors:** Amelia Gulliver, Alison L Calear, Matthew Sunderland, Frances Kay-Lambkin, Louise M Farrer, Michelle Banfield, Philip J Batterham

**Affiliations:** 1 Centre for Mental Health Research Research School of Population Health The Australian National University Canberra Australia; 2 The Matilda Centre for Research in Mental Health and Substance Use University of Sydney Sydney Australia; 3 Priority Research Centre for Brain and Mental Health University of Newcastle Newcastle Australia

**Keywords:** mental health, internet, anxiety, depression, technology, treatment adherence and compliance

## Abstract

**Background:**

Self-guided web-based mental health programs are effective in treating and preventing mental health problems. However, current engagement with these programs in the community is suboptimal, and there is limited evidence indicating how to increase the use of existing evidence-based programs.

**Objective:**

This study aims to investigate the views of people with lived experience of depression and anxiety on factors influencing their engagement with self-guided web-based mental health (e–mental health) programs and to use these perspectives to develop an engagement-facilitation intervention (EFI) to increase engagement (defined as both uptake and adherence) with these programs.

**Methods:**

A total of 24 community members (female=21; male=3) with lived experience of depression and anxiety or depression or anxiety alone participated in 1 of 4 focus groups discussing the factors influencing their engagement with self-guided e–mental health programs and the appearance, delivery mode, and functionality of content for the proposed EFI. A subsequent evaluation survey of the focus group participants (n=14) was conducted to evaluate the resultant draft EFI. Data were thematically analyzed using both inductive and deductive qualitative methods.

**Results:**

Participants suggested that the critical component of an EFI was information that would challenge personal barriers to engagement, including receiving personalized symptom feedback, information regarding the program’s content or effectiveness and data security, and normalization of using e–mental health programs (eg, testimonials). Reminders, rewards, feedback about progress, and coaching were all mentioned as facilitating adherence.

**Conclusions:**

EFIs have the potential to improve community uptake of e–mental health programs. They should focus on providing information on the content and effectiveness of e–mental health programs and normalizing their use. Given that the sample comprised predominantly young females, this study may not be generalizable to other population groups. There is a strong value in involving people with a lived experience in the design and development of EFIs to maximize their effectiveness.

## Introduction

Common mental disorders such as depression and generalized anxiety disorders are experienced by 5% to 10% of the population each year [1-3]. Depression and anxiety can cause high levels of disability and burden [4,5]; however, only one-third of those experiencing a disorder seek professional help [6]. Mental health programs delivered on the web (*e–mental health programs*) have been proposed as a lower-cost alternative to face-to-face therapy [7]. Web-based programs may be particularly appropriate for those identified as at risk for mental health problems or those with mild-to-moderate symptoms [8]. These programs are evidence-based, often as effective as face-to-face therapy, and have the potential to lessen the impact of many of the key barriers to seeking professional help, including cost, stigma, and accessibility [9].

### Barriers to Uptake and Adherence for eMental Health Programs

Despite addressing some of these critical barriers, community uptake of e–mental health programs is low [10,11]. Studies based on primary care have reported rates of uptake between 3% and 25% [12]. Factors such as awareness of e–mental health programs and community views on the effectiveness of web-based versus face-to-face therapy could be fueling this lack of uptake [13]. Accordingly, research has demonstrated a preference in the community for face-to-face therapy over e–mental health programs [11,14,15]. However, studies have also shown that most people are willing and very few people would refuse to try an e–mental health program [16,17]. Thus, it is important to determine the factors that could explain the lack of uptake in community settings. Overcoming these implementation barriers is critical to gaining the maximum benefit of e–mental health programs in the community. Public campaigns have previously been used to raise awareness of e–mental health programs [18]. However, there is a paucity of evidence regarding the effectiveness of these campaigns and other effective strategies for increasing uptake of e–mental health programs.

Reported barriers to the uptake of self-guided e–mental health programs include a lack of general education, cost of hardware and internet access, and time demands [19]. Other potentially modifiable barriers include acceptability of web-based programs and knowledge of how to use technology [19]. Further research has investigated the issue of *acceptability* barriers to e–mental health programs [12]. The acceptability of these programs is thought to be impacted by a wide range of attitudes, including concerns about data security, anxiety about the internet in general, belief that the programs will not work, poor attitudes to help-seeking in general, a general lack of knowledge about web-based programs, or concern that the programs are not endorsed by health care authorities [9,12,14,19-22].

Poor adherence is also a common feature of self-help e–mental health programs. Only about 56% of users completed their assigned web-based program in trial settings compared with 85% in face-to-face settings and 65% in guided internet-based cognitive behavioral therapy for depression [19]. Adherence is even more problematic in naturalistic settings [23]. One study indicated that only 1 in 3 community users completed a minimum of one module with only 10% of the users completing at least two modules out of 6 [24]. Reasons for poor adherence are complex as the drop outs may be because of the lack of need (eg, healthy users), symptom remission, lack of response to treatment, lack of engaging or relevant content, or a high level of symptoms that can interfere directly with the ability to engage (eg, through low motivation or fatigue) [23-26]. Together, these factors act as barriers to the widespread adoption of potentially effective e–mental health programs, limiting the ability of these technologies to deliver on their potential.

### Engagement-Facilitation Interventions

This study adopts a model of engagement [27,28] that includes both the initiation of the program (uptake) and its continued use (adherence). Research on the theory of planned behavior [29] suggests that if the factors affecting both uptake and adherence in e–mental health programs are addressed, we might be able to increase the overall *engagement* with these programs. Acceptance-facilitation interventions (AFIs) have been described as brief programs to increase acceptance of e–mental health programs [12]. Using the theory of planned behavior, the goal of AFIs is to alter subjective societal norms surrounding the knowledge and use of e–mental health programs [12,30]. Engagement-facilitation interventions (EFIs) are related to AFIs. AFIs aim to increase *acceptability* of internet interventions among end users [12,31]. In contrast, EFIs aim to increase *engagement*, which incorporates both uptake and adherence, by addressing factors associated with the acceptability of internet interventions and additional barriers to engagement, such as a lack of time or perceptions that the benefits of the program are not worth the investment [26]. One study found that the acceptability of e–mental health programs for depression in a primary care setting increased after watching a video-based EFI [12]. However, another study using a video-based EFI for increasing engagement with a chronic pain intervention did not demonstrate increased engagement (uptake or adherence) [32]. It was argued that this failure to find an effect may have been because of the sample’s overall high level of motivation before the intervention [32]. Thus, there remains limited evidence on the effectiveness of EFIs in increasing engagement with e–mental health programs in the community. In addition, although previous studies examining barriers to engagement exist [16,26,33], very few qualitative studies with consumer groups have been conducted, and there is a paucity of research investigating the factors that facilitate the use of self-help e–mental health programs for common mental disorders.

### Consumer Involvement

There is widespread acknowledgment of the importance of partnership in health and medical services with consumers who are defined as people with lived experience of the health condition of interest [34]. Effective involvement in research can be described as that which is appropriately chosen for the task and the skills of both the *involvers*, in this case, the researchers, and the *involvees*, the consumers [35]. It is noted that, at a minimum, consultation with the target population in the creation of a service is critical as it allows for tailoring and an assessment of the appropriateness of the content, which can also improve the uptake of and engagement with services [36].

This study brings together the latest evidence in e–mental health program development and implementation and investigation of the primary factors influencing the engagement of self-guided e–mental health programs for consumers living in the community. We define engagement as addressing both the uptake and adherence to these programs. This study presents the development and preliminary evaluation of an EFI based on the results of a series of consumer focus groups. The resultant EFI will be tested in a randomized controlled trial [37] to assess its effectiveness in improving engagement with an existing e–mental health program.

### Objectives

The aim of this study is to investigate factors influencing engagement (uptake and adherence) in e–mental health programs in a community-based sample of those with lived experience of depression and anxiety. The aim of generating this material is to inform the development of a brief EFI for a specific e–mental health program. The principles identified in the development process may be used to guide the development of a variety of future EFIs to maximize uptake and impact.

## Methods

This study adheres to the Standards for Reporting Qualitative Research reporting guidelines [38]. These are presented in Multimedia Appendix 1.

### Ethical Approval

Approval for the ethical conduct of the study was granted by The Australian National University Human Research Ethics Committee (ANU HREC 2018/257).

### Participants

Table 1 presents the demographic characteristics of those who participated in the focus groups and the evaluation survey. The focus group participants were 24 community members aged between 18 and 70 years, who were identified as having lived experience of depression and anxiety or depression or anxiety alone. Approximately 60% (14/23) of these members participated in the evaluation survey.

**Table 1:** Participant demographic information for the focus groups and subsequent evaluation survey.

Demographic data		Focus groups (n=24)	Evaluation survey^a^ (n=14)
**Age (years)**			
	Mean (SD)	27.9 (12.4)	31.6 (15.3)
	Range	18-70	18-70
**Gender, n (%)**			
	Female	21 (87)	14 (100)
	Male	3 (12)	0 (0)
**Study status, n (%)**			
	University student	15 (62)	9 (64)
	Nonstudent	9 (37)	5 (36)

^a^The survey participants were a subset of the focus group participants.

### Recruitment

We recruited participants through advertisements posted in various local community and university Facebook groups and via direct email to local consumer and caregiver groups in the Australian Capital Territory. We specifically sought to sample a group of relevant consumers who would be a natural target audience for an e–mental health program; this included targeting a sample of young people via a university Facebook group.

#### Inclusion Criteria

To ensure that the group was relevant to the development of the EFI, inclusion criteria listed the characteristics of our targeted e–mental health program users. Participants were required to be adults (aged 18 years or above) who self-identified as having a lived experience of mild-to-moderate depression or anxiety, with no severe distress or suicide plan at present. Given the small number of participants, minimal demographic information was collected to reduce the risk of participants being identified.

### Procedure

#### Focus Groups

We conducted 4 focus groups in July 2018 in Canberra, Australia. A total of 24 adults participated across the 4 focus groups (*n*=5, 8, 6, and 5), which ran for approximately 1-hour each. The groups were moderated by author AG (a lived experience researcher) and an assistant (NK, a research assistant or LF, a mental health clinician). All participants provided written consent to participate in the focus groups and completed a brief demographic survey. Focus group participants were advised of the respectful, voluntary, and confidential nature of the discussion and its purpose. We conducted the groups according to the principles of participatory design and iterative development, whereby the potential users of a product or service are involved in its design [36]. Focus group discussions were digitally recorded and transcribed by a professional transcription service. A research assistant or clinician (NK or LF) recorded field notes during the session. Gift cards of Aus $50 (US $35) were distributed to thank participants for their involvement before the discussion sessions.

### Evaluation Survey

After the completion of the focus groups, the points discussed were used to inform the development of a draft EFI. As a separate stage of the research, we sent an email to focus group participants inviting them to participate in a single focus group to evaluate the resultant draft EFI. The demand for this final group was unexpectedly high with 21 out of 24 participants (21/24, 88%) expressing an interest. To maximize the data collected from this final stage of the research, a web-based survey was offered to collect the participants’ views. Out of the 21 interested participants who were sent a link to complete the survey, 14 (67%) responded. They were sent an e–gift card of Aus $35 (US $25) to thank them for their participation.

### Data Collection

#### Focus Groups

Focus groups were selected as they can capitalize on the interaction among participants to generate richer data than that obtained from individual interviews [39]. However, a limitation of this method is that depending on group dynamics, some participants may find it difficult to voice their opinion. Thus, a modified nominal group technique [40] was used to ensure that all participants’ ideas could be heard. A phase of *silent idea generation* [41] provided all participants with an opportunity to provide ideas for what factors may increase or decrease their engagement in e–mental health programs. This was followed by a group discussion and then an individual ranking activity to determine the relative importance of barriers to engagement, which were previously discussed.

Multimedia Appendix 2 presents the list of focus group questions for each of the 4 groups (groups 1-4). The discussions were highly structured and based on predetermined topics sourced by the authors from previous reviews of the literature [9,12,14,19-21]. Participants were initially provided with a verbal description and visual examples of self-guided e–mental health programs to ensure clear understanding of the topic before the discussion. Overall, 2 key topics were discussed in each group, such as (1) factors decreasing (barriers) and increasing (facilitators) their potential engagement in web-based self-guided mental health programs and (2) preferences for EFI content and presentation (eg, video, text, audio, presenters).

Multimedia Appendix 3 presents the written activities that were also completed by participants within the focus groups. The activities were designed to identify the most critical factors decreasing (barriers) and increasing (facilitators) their engagement in e–mental health programs. Participants were instructed to write, on their worksheet, 3 things that might stop them from engaging in a web-based self-guided mental health program (activity 1, targeting both uptake and adherence) and 3 things that could help keep them engaged in a web-based self-guided mental health program (activity 3, primarily targeting adherence). They were also asked to rank their top 3 barriers to engaging in web-based self-guided mental health programs by numbering them (1, 2, and 3) in the order of the barriers they felt were the most important in stopping them from engaging with these programs (activity 2, targeting both uptake and adherence). Participants in focus groups 2 to 4 were also presented with a basic EFI prototype to comment on, which was iteratively developed based on the discussion and input of previous focus groups.

Relevant probing questions were used to gather further details where necessary. The researcher (AG) conducting the focus groups used their lived experience to develop rapport with the participants and to encourage open responses. The groups were continued until the lead author was satisfied that theoretical saturation of ideas about the factors influencing engagement and design concepts for the EFI had occurred (no new insights generated from the data) [42].

### Evaluation Survey

Multimedia Appendix 4 presents the questions included in the evaluation survey to gauge focus group participants’ opinions on the draft EFI developed as a result of the focus groups. The survey consisted of a range of quantitative and qualitative measures assessing what the participants liked and disliked about the EFI, what was missing from the EFI, the likely effect the EFI would have on their decision to start and complete an e–mental health program, their satisfaction with the EFI, and identifying to whom the EFI may appeal.

### Analytical Strategy

Thematic analysis [43] was used by the first author (AG) to group key concepts into themes. For the ranking activity as part of the modified nominal group technique, the relative importance of the ranked barriers was assessed by reverse scoring the ranks, which were then cumulated across participants and tabulated. Thematic analysis of the focus group discussion on both factors influencing engagement and the proposed presentation of the EFI was primarily deductive. Structured questions that were developed ahead of the focus groups based on previous literature on engagement in e–mental health programs were used to develop themes for key factors for inclusion in the EFI. This was complemented by themes generated inductively from written activities that were used to create thematic concept maps. Data from the written activities are presented first, followed by the focus group discussion on factors influencing engagement, including the proposed presentation of the EFI. Finally, the results of the evaluation survey that evaluated the draft EFI are presented. To protect privacy, participants were identified using a participant number.

## Results

### Focus Group

#### Written Activities

##### Factors Decreasing Engagement

**Figure 1:**
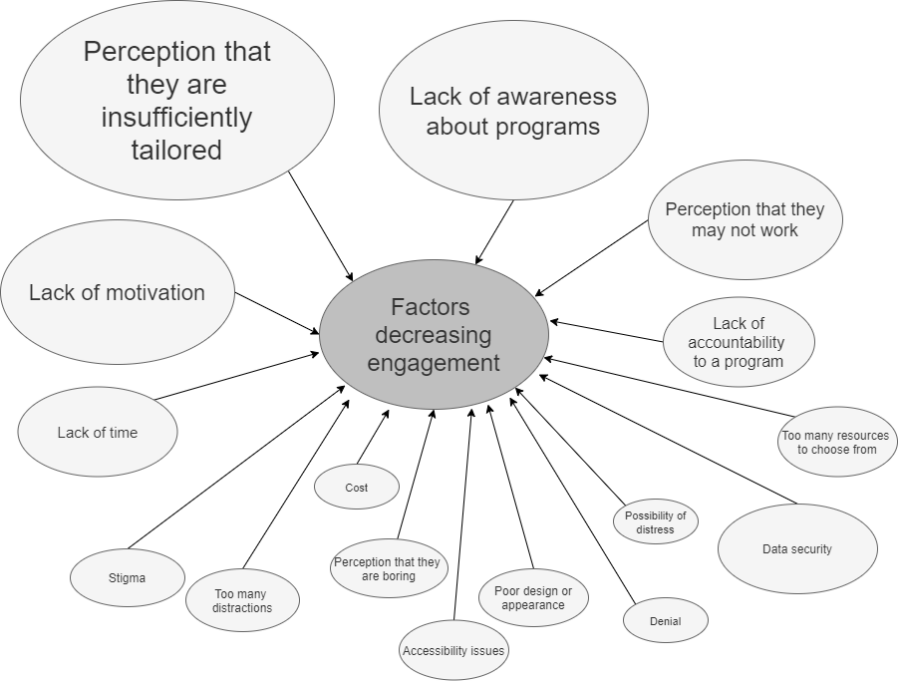
Factors proposed to decrease engagement from the written activity. Larger shapes indicate a greater number of factors reported in that theme.

Figure 1 presents a concept map of the factors that participants identified as likely to decrease their engagement with self-guided e–mental health programs from written activities. The most common factors or barriers to engagement identified were (1) perception that the programs could not be sufficiently tailored or personalized because of their web-based delivery (15/74, 20%), (2) perception of a lack of awareness about the existence of web-based programs (14/74, 19%), (3) perception of difficulties with self-motivation to complete a web-based program (11/74, 15%), and (4) perception that e–mental health programs may not work (9/74, 12%).

##### Ranked Barriers to Engagement

Table 2 provides a summary of the ranked barriers to engaging with an e–mental health program. Overwhelmingly, the most highly ranked factor was related to not knowing whether the program would help them. This was followed by a general lack of awareness of e–mental health programs.

**Table 2:** Barriers to engaging in e–mental health programs as ranked by participants in the written activity.

Rank	Barriers	Score^a^
1	“I don’t know if the online program will help me”	48
2	“I’m not aware of online mental health programs”	19
3	“The online program is too hard to use”	10
3	“I think that I should be able to solve my problems on my own”	10
5	“I’m worried about data security”	9
6	“I’m not comfortable or familiar with existing online mental health programs”	6
6	“I’d be worried about someone finding out I was using an online mental health program”	6
8	“I’m worried that using online mental health programs isn’t something normal, that lots of people do”	3
8	“I don't think any of these would stop me engaging in online self-guided mental health programs”	3
8	“Other (please explain)—User interface design”	3
8	“Other (please explain)—Not understanding or relating to the material, or activity, and knowing if you did it correctly”	3
8	“Other (please explain)—Less accountability to engage than face to face”	3
8	“Other (please explain)—Too many options (overwhelming)”	3
14	“Other (please explain)—They do not present an accurate picture of my mental health”	2
14	“Other (please explain)—No feedback/validation”	2
14	“Other (please explain)—The amount of effort to use it”	2
14	“Other (please explain)—Feeling that face-to-face mental health help is more useful”	2
14	“Other (please explain)—I feel I need a therapist”	2
19	“Other (please explain)—Don’t know what the program entails/what to expect”	1
19	“Other (please explain)—They make me feel more depressed because it reinforces that there is no one there who cares (wants to talk to me)”	1
19	“Other (please explain)—Lacking the motivation to access the program”	1
19	“Other (please explain)—Expecting that there will be a lot of effort involved”	1
19	“Other (please explain)—I do not want to put in the effort to a potentially dull process”	1
24	“I feel anxious about using the internet overall”	0

^a^Ranks are reverse scored and cumulated across participants. Scores for each topic were calculated by cumulating the reverse-scored ranks (ie, 1=3, 2=2, and 3=1) across participants. Higher scores indicate higher importance.

##### Factors Increasing Engagement

**Figure 2:**
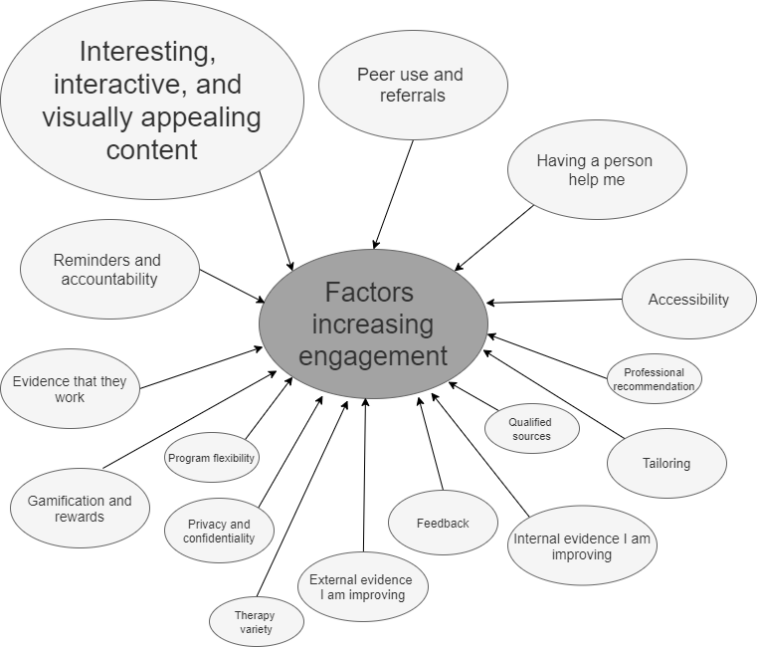
Factors proposed to increase engagement from the written activity. Larger shapes indicate a greater number of factors reported in that theme.

Figure 2 presents a visual depiction of the factors considered to increase participants’ engagement with e–mental health programs from the written activities. The most common factors or facilitators of engagement included (1) a need for the content to be *interesting and engaging* (15/71, 21%) and (2) the knowledge that *peers had used the program* (9/71, 13%). Despite being specifically asked about self-guided programs, several participants (8/71, 11%) indicated a need for outside input from a mental health professional such as a psychologist. A smaller number of comments suggested that the program needed to be easily accessible (eg, works on phones; 5/71, 7%), and some comments expressed a desire for gamification and rewards (4/71, 6%).

#### Focus Group Discussion

##### EFI Development

Overall, participants identified 2 main concerns that needed to be addressed in an EFI to increase user engagement, including both uptake and adherence to a self-guided e–mental health intervention. These were information about (1) what the program involved and what they could expect (in terms of activities, time commitments, etc) and (2) proof of the program’s effectiveness from both scientific (information) and peer (*testimonial*) sources. Furthermore, desired components included feedback about symptoms and information about privacy and confidentiality. Participants also provided suggestions on the style and presentation of the overall EFI, noting a strong preference for a simple linear click-through format rather than having the content presented on a single page. Participants are referred to by numbers (ie, participant number 1=P1).

##### Key Factors for Inclusion in an EFI

###### Awareness

Most participants were not aware of the e–mental health programs that we presented as examples in the focus groups. This lack of awareness was noted by participants as a key factor that reduced e–mental health program uptake in the community*.* Although e–mental health programs were considered beneficial for help-seeking, particularly for those who felt uncomfortable with face-to-face care, participants felt, “you have to know of its existence [first]” (P1).

###### Information About What the Program Involves

Overall, participants reported a strong need for an EFI to contain information about what would be involved in completing the subsequent e–mental health program. This included descriptions of the therapy type and the activities they would be required to complete. One participant remarked that they wanted to know about the specific therapy types (eg, cognitive behavioral therapy). Before participating in a program, participants also wanted to know how structured or prescribed the overall program would be, the frequency and duration of recommended usage, and also the number of sessions or timeframe before they could expect to feel better. One participant thought that it would be motivating to display the potential for symptom improvement on a graph—“here’s where you are and with use of our program we predict you could be all the way down to here” (P2).

###### Evidence of the Program’s Effectiveness

To increase the likelihood of starting and continuing to use a program, participants also reported the need of evidence of a program’s effectiveness based on both scientific sources and peer reviews or testimonials. Many participants reported a desire for evidence in the EFI that a web-based program works for people “like them” or with similar psychological problems. They also wanted to know from the EFI that the e–mental health program was tailored and could adapt to each person, that it could handle more than one problem at once (comorbidity), and that it was worth the time investment:

When you’re feeling a bit low or something it takes extra effort to go in and do something and if you’re going to spend a lot of your limited energy on something and you don’t think there’s going to be of a difference from where you are now then you might not bother with it if it’s not very effective.

P3

It was considered particularly important for increasing uptake and adherence for an EFI to demonstrate the effectiveness of web-based programs specifically, as participants noted that there remains, in the community, strong “perceptions that [e–mental health programs are] not as effective as face-to-face” (P4).

Participants also suggested that the EFI could include evidence of effectiveness from peers who had used the program. It was a common view expressed across all 4 groups that multiple and varied genuine testimonials (*reviews*) were desired as further *proof* that the program worked. Viewing a review from someone they trust who has had success with the program was considered as, or even more, important than evidence from scientific or professional sources. However, these reviews had to appear genuine, as participants believed that they could tell if they were faked—usually they were viewed as disingenuous if the reviews were too positive or if there were no negative reviews. In addition, if the people in the reviews were too attractive or not sufficiently varied in appearance, these were also viewed as simulated:

There needs to be a variety. I think you can’t just have textbook 30 something woman, you know, aesthetic face. I think there needs to be a variety of ages because I think these things need to cater to older generations as well as younger ones. And also a variety of ethnicities I think is very important…They can’t all be beautiful.

P5

The participants also wanted reviews to take into account complex and relevant issues such as outlining what is different, better, or worse than face-to-face therapy.

###### Other Concepts

Several other topics were also discussed to a lesser degree, including the provision of feedback, privacy, and confidentiality information, emergency contacts, and cost. Some participants believed that it was important to provide feedback on symptoms, particularly before starting a web-based self-guided mental health program to help them accept that they needed help. They also thought that it would be helpful if the EFI could provide a description of what type of mental health problem they likely had and evidence that a program could be effective for and tailored to that particular type of mental health problem. Some participants wanted financial information to be included in the EFI about how much it would cost them personally to use the program, as this was considered as important information in their decision to start the program. Other information desired was how privacy and confidentiality would be maintained. Some believed privacy was the most important factor for deciding whether to engage in these programs; whereas, many others felt that it was now part of modern day life to give up some privacy to access web-based or mobile services and that “this is just the way the world is now, everyone knows everything...my phone’s listening to me right now” (P6).

However, this did not extend to all information; there was a preference for providing less personal information:

It doesn’t worry me that much. Particularly ‘cause I’m not putting in my super, super personal information. Yeah, if I was...write down my address and you know the names of these people that you’ve seen and your GP, and whatever, maybe that would worry me more.

P7

Conversely, participants with concerns relating to their place of employment shared that they may not start an e–mental health program without evidence that the program was completely confidential and that their data would be kept private.

##### Optimal Presentation for the EFI

Participants also provided advice about the overall style and presentation of the EFI, as this would also impact their likelihood of engaging in a program.

###### Overall Presentation and Delivery

In general, participants did not like the content of the EFI being displayed statically on one page; they found it overwhelming and confusing. They commented that they would prefer to have information presented slowly and step-by-step, citing that when experiencing symptoms of anxiety or depression, too much content or words on a page could be stressful. In addition, for similar reasons, all but one participant also disliked the idea of one page with the inclusion of drop-down menus to access content.

###### Presentation of Symptom Feedback

Participants were shown multiple versions for how feedback on symptoms could be presented in an EFI after the completion of a brief assessment of depression or anxiety: a traditional histogram-style graph, a traffic light, or a simple meter pointing to the symptom severity. In general, participants preferred the simplicity of a meter with 3 or 4 points to display the severity of symptoms. However, they did say that a small amount of explanatory text was essential so that they could understand what the feedback meant. However, some of the participants in one group did not like cutoffs for the meter, preferring a *spectrum* model of symptoms:

I like things that kind of have kind of a spectrum, which I think both of these do. Because…if you draw a line then that is really stressful for people who are on one side or people who are kind of getting towards that line.

P6

Some participants preferred the more complicated histogram-style graph, although they explicitly noted that they did not want to be presented with normative population data. They noted that they already knew how bad they felt and comparisons with other people would likely make them feel worse, particularly if their symptoms appeared more or less severe than the population or were incongruent with how they felt:

Yeah, I don’t really want to know everyone else’s because I’m sure everyone else was probably a lot better than mine and that makes me feel really ostracised…I really am depressed and everyone else is so far ahead so what’s the point in trying? It would just make me feel worse

P8

###### Presentation of Information

Overall, text was the dominant preference for the display of information in an EFI; however, it was clear that this text had to be brief and preferably with bullet points for ease of reading. Participants believed that video presentation in an EFI may be preferred by some users, although, again, they indicated that it must be brief (ie, 30 seconds or less). They also said that the videos should be accessible, including subtitles or a written transcript. Suggestions for presenters within an EFI video were very broad—some participants wanted someone famous who could elicit trust, such as a well-known athlete, some wanted experts, and some mentioned that they would prefer animations or cartoons that transcend specific population groups. A strong preference for variety in the way that information was presented in an EFI emerged, and several participants mentioned that a mixture of presentations would likely help to keep them engaged. Variety was also preferred for flexibility of use, such as using audio with headphones or preferring visual data on public transport. When asked if they wanted a helper or a *guide* character to accompany them through the EFI, some participants agreed that this could be helpful, but they explicitly noted that it must be optional as “you want to encourage people to look at it and click on it, you don’t want it just in their face” (P3).

###### Presentation for Testimonials and Reviews

When participants were asked about their preferred delivery mode for the testimonials they desired in the EFI, there was a strong preference for video as it was easier to judge whether they thought that it was real or not, and it was more believable and likely to be genuine because of the higher degree of effort involved compared with written forms. Overall, many participants stated that they wanted to see as many different testimonials as possible. This included testimonials from different genders, ages, and ethnicities so that the participants could find the one they identify themselves with as “if they’re a completely different person to me, I’m just not sure if I could relate to them and their experiences” (P9).

### Evaluation Survey

The draft EFI shown to the participants in the evaluation survey is presented in Multimedia Appendix 5. Table 3 presents the quantitative data collected in the evaluation survey assessing the focus group participants’ views on the resultant draft EFI. Overall, participants reported that the draft EFI would likely have a modest effect on their uptake and engagement with e–mental health (*myCompass,* developed by Black Dog Institute). Participants were also satisfied with the EFI and the way it captured their suggestions, and they were satisfied to highly satisfied with their participation in the study.

**Table 3:** Participant satisfaction of the engagement-facilitation intervention created via the focus groups.

Question item	Participants, n	Score, mean (SD)
“What effect would the EFI^a^ have on your decision to start using myCompass?”	14	4.07 (0.83)^b^
“What effect would the EFI have on your decision to complete myCompass?”	14	3.93 (0.48)^b^
“How satisfied are you with the EFI overall?”	13	4.08 (0.64)^c^
“How satisfied are you with the way the EFI has captured your suggestions?”	13	4.08 (0.49)^c^
“How satisfied are you with your participation in this study?”	13	4.54 (0.52)^c^

^a^EFI: engagement-facilitation intervention.

^b^Scored as 5=much more likely, 4=a little more likely, 3=no change, 2=a little less likely, and 1=much less likely.

^c^Scored as 5=highly satisfied, 4=satisfied, 3=neutral, 2=dissatisfied, and 1=very dissatisfied.

#### Participants’ Views on the EFI

Participants reported approving of a number of aspects of the draft EFI, including the brief, easy-to-read information that effectively used bullet points and headings to aid understanding. Participants also reported enjoying the video testimonials but requested that there should be a greater variety and number of them. Half (n=7) of the participants praised the design, graphics, and color scheme as being “engaging but doesn’t distract from the information provided” (P10), whereas 2 participants mentioned that they did not like the color scheme—the orange in particular. Finally, several participants liked the graphical feedback and the impression that it was professional, tailored to the user, flexible, and provided recommendations.

Participants were also invited to report what aspects of the EFI they did not like. A number of participants (n=5) had no comments for this question—“I like it as it is. I was actually feeling ready to get started!” (P11), whereas others reported very specific suggestions about adding clearer information about the potential cost (or the lack thereof) and the removal of a line at the end of the feedback for depression and anxiety that sought rhetorical confirmation from the participant (eg, “Does this sound right to you?”). The participant noted that this question sounded tentative and unlikely to provide a sense of confidence in the program. These suggestions were used to change the final version of the EFI for the RCT.

#### Potential Influence of the EFI

Several participants noted that the EFI was likely to influence them to start the e–mental health program, with one participant indicating that “the statement that it could be as useful as medication would be very persuasive for me” (P9). However, participants disagreed on whether the time required to use the program seemed too long. Some said that the recommended timeframes, for example, 60 min to 90 min, and 15 min per module seemed onerous. Others thought that a 15-min block of time to complete a module was easily digestible. Another participant acknowledged that although the time period seemed intimidating as a commitment, they believed that the addition of the word *recommended* helped them to overcome this barrier to uptake. Several participants noted that they found the EFI presentation and content simple to read and made the e–mental health program seem achievable. Another participant also mentioned that the EFI was not overwhelming and that this was critical to them to be able to do a program while experiencing mental health problems:

I wanted to start using myCompass now. I was really surprised, because I dislike online therapy programs. The EFI makes it…a fun activity rather than therapy, and one which would appeal to me when I was depressed, rather than being hard work. When I am depressed it is difficult for me to do any activities, so anything that appeals to me makes it easier to do. The EFI makes it the opposite to overwhelming. When I am depressed, I am overwhelmed.

P12

Finally, one participant noted that the program was unlikely to encourage her to use it, given her preexisting privacy concerns about using such a program.

#### Groups to Which the EFI Would Appeal

A number of participants reported that the EFI looked as though it was targeted at younger people or adults (n=7), possibly because of the bright colors used and the fact that it was delivered via the internet. However, several other participants noted that it did not seem to be targeting any specific demographic, including age and gender, which they found appealing.

## Discussion

### Principal Findings

This study presents a detailed examination of consumers’ views of factors influencing their potential engagement with self-guided e–mental health programs for depression and anxiety. In addition, it provides consumer perspectives on the development of an EFI to improve engagement with these programs. Overall, this study indicates that presenting multiple forms of evidence that an e–mental health program can be effective was seen as the primary driver of whether an EFI could improve the uptake and engagement of the e–mental health program. A critical factor preventing participants’ uptake of e–mental health programs was uncertainty about whether it could help them and whether it is worth investing their time and energy. This is consistent with previous research identifying low expectations of effectiveness as an important barrier for the uptake of e–mental health programs [12]. The information needed to confirm this varied, including demonstrating that it was sufficiently tailored, showing that other people used it via testimonials or peer use, and evidence for the effectiveness of web-based programs, which have been found to be important factors for the uptake of e–mental health programs previously [10,21]. In particular, participants reported a strong desire to receive information about program effectiveness from trusted peers. This is congruent with the theory of planned behavior, noting the importance of subjective norms in changing an individual’s actions [29]. It was noted as important that these people were believable and real. However, consistent with previous research, participants also wanted scientific evidence in addition to evidence presented by peers [16]. It is likely that this issue can be addressed in part by the provision in an EFI of information about effectiveness, what it involves, what they can expect by participating, and testimonials. The participants’ views on videos from this study were consistent with previous research [44] where videos had been found to be the most effective form of providing testimonials. Testimonials can contain powerful messages by using identification with the person telling the story and the viewer can relate and empathize with the person [44]. Testimonials have also been found to increase belief and message uptake more than the presentation of statistical data [45].

Privacy was not considered an important factor by most participants in influencing e–mental health program usage in this study. This differs from previous studies that had found that security and confidentiality of personal information concerns are considered critical for using e–mental health [16,22]. However, certain people in the community may have a greater desire for privacy and confidentiality assurances, which highlights the importance of clearly providing this information to ensure that participants are not opting out based on its absence. Concerns about privacy are also likely to depend on how much information is sought within a digital intervention; anonymous programs are likely to cause less concern than a program requiring names, email addresses, or phone contacts.

The finding that participants did not want to see their scores displayed in the EFI relative to normative population data was unexpected, particularly because the presentation of normative data is commonly used to motivate help-seeking for multiple mental health problems, including alcohol use [46] and depression [47]. The idea behind presenting normative data is to reduce normative misperceptions about alcohol or other drug use [48] and to motivate the person to seek help. However, it is possible that this does not translate directly to other mental health problems and that there may be sensitivities and stigma associated with labeling an individual as having a certain level of symptoms. An approach taken in the alcohol or other drug usage field when presenting normative feedback may be important in this context, that is, permission is sought from the participant before providing normative feedback, giving the person some control over the type of feedback to which they are exposed. Some participants did find the concept of graphically presenting their baseline score and their capacity to improve with treatment as important, suggesting that if feedback is provided, it may need to be in the context of messaging how (and by how much) symptoms can be improved by engaging with the e–mental health program. Potentially, an algorithm for future EFIs could be presented to people about to embark on an e–mental health program that uses average improvement scores drawn from previous research with that program to estimate the percentage symptom improvement a participant could expect based on their baseline score. However, it is important to note that it is difficult to convey the complexity of changes in severity over time using a simple illustration, so there may be unintended consequences of presenting this information, such as a sense of *failure* if expected gains are not achieved. More research on this particular issue is warranted.

There was a significant lack of awareness of available e–mental health programs among participants. Given that recruitment specifically noted the topic, this was an unexpected finding. Despite local public campaigns about e–mental health programs [18], it appears that there may be a lack of knowledge in the community about these programs. As noted by both Ebert et al [12] and Gun [21], an alternative avenue of increasing knowledge in the community about the availability and effectiveness of e–mental health programs is to better inform clinicians about e–mental health programs, as they are important gatekeepers to the usage of these programs [49]. However, engaging clinicians may be a complex process, and most people with mental disorders do not engage with mental health professionals [6]; thus, direct-to-consumer pathways are also needed to maximize the impact of e–mental health programs in the community [9,50].

### Implications for Intervention Development

Consumer views are critical in the development of new interventions and resources [36]. However, it is important to recognize that perspectives often vary broadly and it is often not possible to reach group consensus [51]. In the creation of an intervention, it is important to balance the selection of components using information that combines both the best available evidence (eg, content that aligns with theory, previous research, consumer views) with the practical constraints of the intervention development. For example, it was not feasible for the EFI that was developed in this study within our budget to create multiple testimonial videos of a wide variety of genders, ethnicities, and ages. However, on balance, this factor was also not deemed to be as critical or as practical to implement as education about how e–mental health can help people in the community. Thus, a pragmatic approach to the development of interventions using consumer perspectives is recommended. Broadly, evidence from this study suggests that EFIs should include information about the purpose of e–mental health programs and its components and evidence demonstrating that the program is effective. Use of video testimonials, simple text and images, and simple feedback about symptoms are also likely to be useful.

Increasing consumer engagement with internet interventions is challenging. However, ensuring that the content is interesting, interactive, and visually appealing may increase engagement—color selection and the inclusion of graphics is particularly important. Despite explicitly examining self-guided interventions, several participants noted the need for accountability to *someone*. Having a virtual guide assist the users through a program may address the barrier of a lack of motivation to engage by addressing the perceived lack of accountability to a computerized program (as opposed to a therapist). Dynamic engagement processes through the course of an intervention, such as the use of guides and reminders, may be more useful for promoting adherence than passive information provided at the start of the intervention.

Knowing peers who had used the program or providing a referral was seen as helpful. Testimonials may provide a proxy for personal referral; however, they were seen by some to be *fake* or less compelling. This issue could potentially be addressed by the inclusion of authentic video testimonials that explicitly address potential challenges with the program, particularly by avoiding the use of actors. Ensuring that the content appears sufficiently tailored to the person is also important. Although it is difficult to ensure that all mental health problems are covered, simple tailoring strategies, such as choice of a relatable avatar, may provide a sense of agency. Finally, to use the programs, the participants said that they had to know that such programs existed. The cost of advertising may preclude large-scale campaigns. In addition, viral campaigns to increase awareness of internet interventions on social media may be considered. When delivering internet interventions in a community setting, inclusion of these features may lead to greater uptake and adherence.

### Limitations

There were many limitations to this study. The sample was limited to a single state in Australia, with predominantly highly educated (university students) participants. In addition, overall, very few males participated, with none responding to the EFI evaluation survey. However, this is common in other self-selected samples for studies on anxiety and depression [52] where females are overrepresented. In addition, the sample reflects similar gender imbalances in the usage of e–mental health programs in the community, where substantially more females are found to use these services [24]. Engaging men in mental health research is challenging; they may be less likely than women to participate because of specific factors such as stigma [53]. Despite this, these issues with the sample limit the generalizability of the study to other populations. It is also possible that the evaluation survey could have been biased toward those who were more engaged in the process, and thus, this may not be reflective of the entire sample. In addition, many participants stated that they believed the resultant EFI would increase their engagement with a web-based self-help e–mental health program; however, this may not reflect their actual behavior.

### Conclusions

One of the critical barriers to the uptake of e–mental health programs is the lack of certainty among community members about whether the program can help them. A second major barrier to uptake is the lack of awareness of e–mental health programs and their availability in the community. This study found that an EFI for depression and anxiety primarily requires information about what the program involves, such as evidence for its effectiveness, normalization of participation in e–mental health programs, including testimonials, and finally, brief information on data security, although this factor was not as prevalent as expected. Attention to these factors may guide the development of future technology-based interventions that are designed to increase engagement and adherence.

## Appendix

Multimedia Appendix 1 Reporting checklist for qualitative study.

Multimedia Appendix 2 List of questions.

Multimedia Appendix 3 Written activities.

Multimedia Appendix 4 Questions from online follow-up survey.

Multimedia Appendix 5 Draft EFI presented to participants in evaluation survey.

## Abbreviations

AFI: acceptance-facilitation intervention
EFI: engagement-facilitation intervention
NHMRC: National Health and Medical Research Council
