# Supplementary material for: Consumer-Guided Development of an Engagement-Facilitation Intervention for Increasing Uptake and Adherence for Self-Guided Web-Based Mental Health Programs: Focus Groups and Online Evaluation Survey
Source: JMIR Form Res. 2020 Oct 29;4(10):e22528. doi: 10.2196/22528 (PMC7661236; doi:10.2196/22528)
Supplement: Multimedia Appendix 2 [file formative_v4i10e22528_app2.docx]

## Multimedia Appendix 2. List of questions

**Questions for all Groups**

<presented examples of e-mental health programs.

1. **Activity 1** - Please write down 3 things that might stop you from engaging in an online self-guided mental health program?
2. What sorts of things do you think might prevent people from engaging in online self-guided mental health programs? (omitted for groups 2, 3, 4 for time constraints as these were captured by Activity 1).

Some of these potentially modifiable factors (i.e., acceptability) include: <(go through each individually and ask – would this be a barrier to you in engaging in an online mental health program?)>

- **Expectancies of effectiveness** – Weighing up if you think the program will work or not?
- **Effort expectancies?** How much effort is involved? How easy is it to use?
- Concerns around data security.
- **Not being aware** of online mental health programs.
- **Not feeling comfortable** or **familiar** with existing online mental health programs.
- **Negative attitudes** or **stigma** around help-seeking for mental health problems overall.
- Feeling like you have to have **certain skills** to use the programs, or feelings of anxiety around internet usage more generally (added for groups 2, 3, 4).
- **Social influence** – if you think that no one else is using it. How much do you think other people are using these programs? (added for groups 2, 3, 4).

1. **Activity 2** - Please rank your top 3 barriers to engaging in online self-guided mental health programs below by numbering them (1, 2, 3) in order of those you see as the ***most important*** in stopping you from engaging with these programs.
2. **Activity 3** - Please write down 3 things that could ***help keep you engaged*** an online self-guided mental health program?
3. What sorts of things do you think might help people engage in an online self-guided mental health program? ***Prompt*** – what types of specific information do you think would help you to decide whether to engage in an online self-guided therapy program?
4. We know that there are some components that have evidence for being motivating for people, these include giving people feedback about their symptoms of mental health problems, and education around some of those factors we spoke about before including data security, and helping people understand that these programs can be very effective. What mode of presentation do you think might work best for providing this information? Visual examples provided of:

- Feedback – graphs, text, traffic lights (*Q: Which graph and explanation do you prefer, and why? Are there any drawbacks to either graph? - added for group 4).
- Education – text, bullet points, infographics, video, audio (*Q: If there was a video to teach you how to use the site - Would you prefer this information to be delivered by a health professional/researcher, a cartoon, or a person who had used the site? - added for group 4).
- Testimonials – videos, audio, cartoon, text). ***Prompt*** – For educational videos - what sort of presenter do you think would be most trustworthy or believable – would you want a researcher or clinician? Or someone with lived experience or a celebrity? What about an animated character? And do you think age, gender etc. what they look like matters? ***Prompt*** – Would this be the same or different for testimonials? What type of person or character would you want for that? (*Q: For **educational videos** – most people in our groups have said that they prefer videos from real people, as it makes them seem more genuine, and it’s harder to fake. How many do you think we would need to get a diverse enough group? - added for group 4).

1. If you were given some feedback about your own level of mental health symptoms and it suggested that you try an online mental health program – how credible would you find those suggestions?

**Additional questions for Group 2**

What we are trying to do today is try to provide something like a mini-educational intervention or website that will help people understand more about what an online program is all about, and hopefully help them be more motivated to start it, and also to complete it.

This is our little non-functional prototype with a made up program called **Health Online** that we’ve created based on some feedback we got in the first focus group.

So you can see that some of the components included are

- Feedback about symptoms for anxiety and depression in visual form, and afterwards dot points about what this means, and what you can do about it.
- Some information about data security and how we keep your information safe.
- Some links to evidence that the program works.
- Some information about what the program involves – and how long it will take etc.
- And perhaps some videos of people who have used the program, and what it’s done for them.

1. First of all – what are your first impressions of this content, (keeping in mind it won’t visually look like this)? Is there anything missing?
2. How would you feel with all of this being on the one page? ***Prompt:*** We are a bit concerned that if we have multiple pages, we might lose people, so making it all interactive, with drop downs, videos, and on the one page might be more interesting? Overwhelming?
3. Would you be happy with the mode that things are presented? Or would you prefer, say videos, for the information rather than text, or testimonials in writing?
4. Would something like this have broad appeal? What groups in the community do you think this might appeal to/ not appeal to?

**Additional questions for Groups 3 and 4**

What we are trying to do today is try to provide something like a mini-educational intervention or website that will help people understand more about what a specific online program is all about, and hopefully help them be more motivated to start the program, and also to complete it.

I’m going to show you some non-functional prototypes with a made up program called **Health Online** that we’ve created based on some feedback we got in the first two focus groups.

So in this example, a person would complete a couple of short quizzes about their symptoms of depression and anxiety, and then would be presented with some information designed to increase their motivation to do a specific online program.

I’m going to show you a couple of different ways we could present this information – the first is in a linear way –

So you’ll see that some of the components included are *<click through in linear way>:*

- Feedback about symptoms for anxiety and depression in visual form, and afterwards dot points about what this means, and what you can do about it.
- Some videos of people who have used the program, and what it’s done for them.
- Some information about what the program involves – and how long it will take etc.
- Some information about data security and how we keep your information safe.
- And some info about evidence that the program works.

1. First of all – what are your first impressions of this content, (keeping in mind it won’t visually look like this)? Is there anything missing?
2. How would you feel with all of this being on the one page?
3. We could also present this on one page in an interactive way where you can click through the content but it’s not all available at once? (show click downs and explain how it would likely look on a mobile – menu options). ***Prompt:*** We are a bit concerned that if we have multiple pages, we might lose people, so making it all interactive, with drop downs, videos, and on the one page might be more interesting? But would it be overwhelming?
