# Supplementary material for: Consumer-Guided Development of an Engagement-Facilitation Intervention for Increasing Uptake and Adherence for Self-Guided Web-Based Mental Health Programs: Focus Groups and Online Evaluation Survey
Source: JMIR Form Res. 2020 Oct 29;4(10):e22528. doi: 10.2196/22528 (PMC7661236; doi:10.2196/22528)
Supplement: Multimedia Appendix 3 [file formative_v4i10e22528_app3.docx]

## Multimedia Appendix 3. Written activities

**ACTIVITY 1** – Before “Barriers” discussion

Please write down 3 things that might ***stop you from engaging*** in an online self-guided mental health program?

1.

2.

3.

**ACTIVITY 2** – After “Barriers” discussion

Please rank your **top 3 barriers to engaging in online self-guided mental health programs** below by numbering them (1, 2, 3) in order of those you see as the ***most important*** in stopping you from engaging with these programs:

**1 =** most important barrier for me

**2 =** 2^nd^ most important barrier for me

**3 =** 3^rd^ most important barrier for me

| **Barrier** |  |
| --- | --- |
| I don’t know if the online program will help me |  |
| The online program is too hard to use. |  |
| I’m worried about data security. |  |
| I’m not aware of online mental health programs |  |
| I’m not comfortable or familiar with existing online mental health programs. |  |
| I think that I should be able to solve my problems on my own. |  |
| I’d be worried about someone finding out I was using an online mental health program. |  |
| I feel anxious about using the internet overall. |  |
| I’m worried that using online mental health programs isn’t something normal, that lots of people do. |  |
| Other (please explain) |  |
| Other (please explain) |  |
| Other (please explain) |  |

I don’t think any of these would stop me engaging in online self-guided mental health programs (tick if this applies)

**ACTIVITY 3** – Before “Facilitators” discussion

Please write down 3 things that could ***help keep you engaged*** an online self-guided mental health program?

1.

2.

3.
