# Supplementary material for: Consumer-Guided Development of an Engagement-Facilitation Intervention for Increasing Uptake and Adherence for Self-Guided Web-Based Mental Health Programs: Focus Groups and Online Evaluation Survey
Source: JMIR Form Res. 2020 Oct 29;4(10):e22528. doi: 10.2196/22528 (PMC7661236; doi:10.2196/22528)
Supplement: Multimedia Appendix 4 [file formative_v4i10e22528_app4.docx]

## Multimedia Appendix 4. Questions from online follow-up survey

1. What do you like about the EFI? (it would be helpful for us if your feedback was specific to aspects of the EFI like the colour palette, the language or wording/ type and amount of text, etc)
2. What do you dislike about the EFI? What specifically would you change about the EFI (if anything)
3. What effect would the EFI have on your decision to start using myCompass? (scale: 5 = Much more likely to use myCompass; 4 = a little more likely to use myCompass; 3 = no change, ; 2 = a little less likely to use myCompass ; 1 = much less likely to use myCompass).
4. Please add any comments about why you feel the EFI would have that effect on your decision to start using myCompass (open-ended).
5. What effect would the EFI have on your decision to complete myCompass? (scale: 5 = Much more likely to complete myCompass; 4 = a little more likely to complete myCompass; 3 = no change, ; 2 = a little less likely to complete myCompass ; 1 = much less likely to complete myCompass).
6. Please add any comments about why you feel the EFI would have that effect on your decision to complete myCompass (open-ended).
7. What sorts of people or groups do you think the EFI would appeal to?
8. What other important content do you think the EFI is missing?
9. How satisfied are you with the EFI overall? (scale: 5 = Highly satisfied, 4 = satisfied, 3 = neutral, 2 = dissatisfied, 1 = very dissatisfied).
10. How satisfied are you with the way the EFI has captured your suggestions? (scale: 5 = Highly satisfied, 4 = satisfied, 3 = neutral, 2 = dissatisfied, 1 = very dissatisfied).
11. How satisfied are you with your participation in this study? (scale: 5 = Highly satisfied, 4 = satisfied, 3 = neutral, 2 = dissatisfied, 1 = very dissatisfied).
12. Please add any further comments about your participation in this study (open-ended).
