# Supplementary material for: Consumer-Guided Development of an Engagement-Facilitation Intervention for Increasing Uptake and Adherence for Self-Guided Web-Based Mental Health Programs: Focus Groups and Online Evaluation Survey
Source: JMIR Form Res. 2020 Oct 29;4(10):e22528. doi: 10.2196/22528 (PMC7661236; doi:10.2196/22528)
Supplement: Multimedia Appendix 5 [file formative_v4i10e22528_app5.pdf]

Multimedia Appendix 5. Draft EFI presented to participants in evaluation survey

⚠ This study has not been approved to GO LIVE as yet.

Enhancing Engagement with Psychosocial Interventions (EEPI)

jac16Nov13@example.com Logout

Home About Terms

Visually appealing content

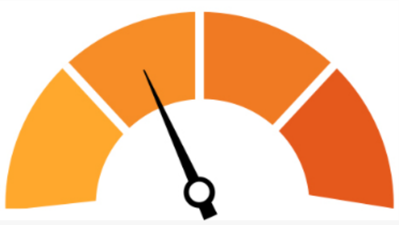

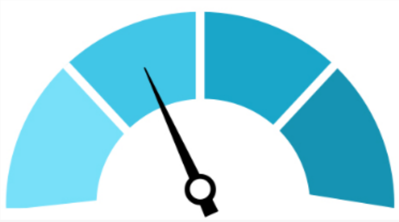

ANXIETY METER

You've just completed a quiz measuring **anxiety symptoms.**

Your score of **5** indicates that you are currently experiencing mild symptoms of anxiety.

Does this sound right to you?

It's normal to experience some anxiety, but when those feelings start to interfere with the things you need to do in your life, or make you feel upset or distressed **it's time to do something about it**.

Psychological therapies are **highly effective** at improving symptoms of anxiety. Starting now is important as it can also help you prevent your symptoms from getting any worse.

The myCompass program is easy to use, and can help you learn effective psychological techniques to **improve how you feel today** and in the future.

DEPRESSION METER

You've also completed a quiz measuring **depression symptoms.**

You've also completed a quiz measuring **depression symptoms.**

Your score of **5** indicates that you are currently experiencing mild symptoms of depression.

Does this sound right to you?

It's normal to sometimes feel depressed or sad, but when those feelings start to interfere with the things you need to do in your life, or make you feel upset or distressed **it's time to do something about it**.

Psychological therapies are **highly effective** at improving symptoms of depression. Starting now is important as it can also help you prevent your symptoms from getting any worse.

The myCompass program is easy to use, and can help you learn effective psychological techniques to **improve how you feel today** and in the future.

Feedback about symptoms

Just a score - No comparison to others (e.g.,graph)

Addressing lack of motivation

Addressing ease of use and potential for improvement

Showing this program can help them.

Next

Black Dog Institute

Trustworthy source with existing reputation

⚠ This study has not been approved to GO LIVE as yet.

Enhancing Engagement with Psychosocial Interventions (EEPI)

jac16Nov13@example.com Logout

Coloured, but neutral imagery (to increase broad appeal

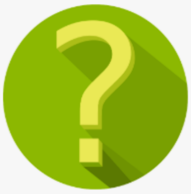

WHAT IS IT?

- myCompass is a **free program** you can use to help improve your mental health.
- myCompass **can help you**, because our test showed that you are experiencing **mild-to-moderate symptoms** of depression, anxiety and/or stress.
- myCompass is private and confidential.

Cost-free

Privacy/data security – link to privacy policy

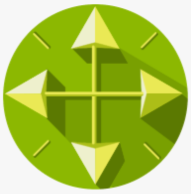

HOW DOES IT WORK?

- myCompass is **tailored to you**. It provides **information and strategies** for challenging unhelpful thoughts and feelings to help you improve your mood and reduce your stress and anxiety.
- Depending on your responses to questions during registration, myCompass recommends online learning modules that will **best match your needs**
- You can also choose to do the **modules you want**, based on your interests.
- myCompass will show you how to track your thoughts, feelings and behaviours and will help you **learn about your own thought patterns and triggers**.

Tailored

Info about content

Tailored

Variety in content

Flexible, tailored to personal preferences

Information about program content

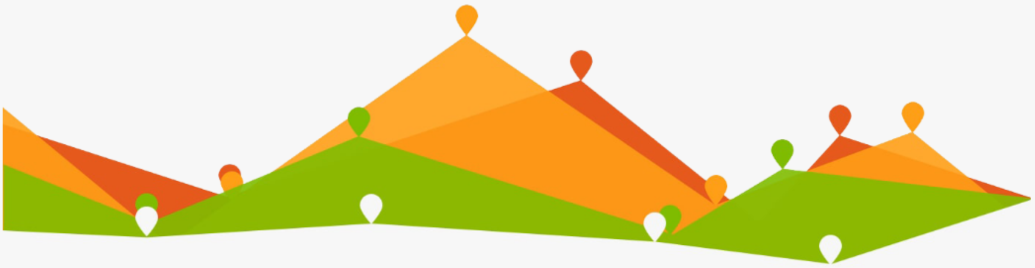

Next

Black Dog Institute

*note – underlined text will link out to evidence.

⚠ This study has not been approved to GO LIVE as yet.

Enhancing Engagement with Psychosocial Interventions (EEPI)

jac16Nov13@example.com Logout

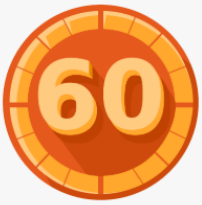

Addresses concerns about time issues

HOW MUCH TIME DOES IT TAKE?

- The program is **flexible**, but you will get the most out of it if you do **two modules each week for 7 weeks (14 total)**. Two modules take about 60-90 minutes.
- The modules are split into **three short 10-15 minute sessions** so you can fit them into your day.
- Mood-tracking only takes around **1-2 minutes per day**.

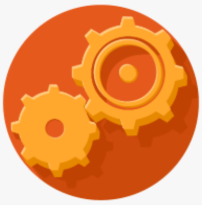

DOES IT WORK?

- myCompass contains proven psychological techniques that psychologists use, such as cognitive behaviour therapy (CBT).
- myCompass can be as effective as antidepressants and can help you achieve longer-lasting and more permanent improvements to mood, anxiety and stress.

Evidence that the program is effective

Challenging lack of e-mental health knowledge - Evidence that it is equivalent to other treatments

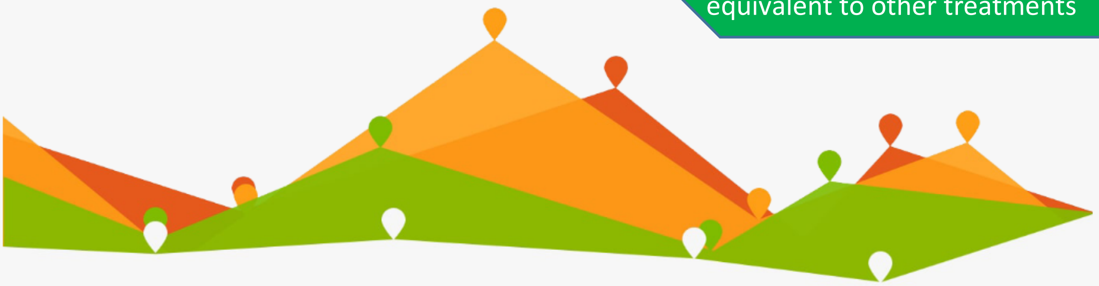

Back Next

Black Dog Institute

*note – underlined text will link out to evidence.

⚠ This study has not been approved to GO LIVE as yet.

Enhancing Engagement with Psychosocial Interventions (EEPI)

jac16Nov13@example.com Logout

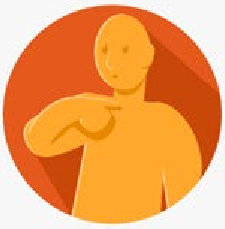

Go to Mycompass

WHY SHOULD I DO IT?

- It works.
- It's **easy** to use
- The tests you took suggest you **may be experiencing some difficulties** with symptoms of anxiety or depression. myCompass is designed to reduce these unhelpful feelings.
- It's **worth your time** to improve your life.
- Online programs and apps are being increasingly used by people in the community to **look after their mental health in their own time**.

More evidence that it works

Ease of use

Worth the "effort"

Addressing societal and peer norms

WHAT PEOPLE SAY?

Accessible transcript

Video Transcript

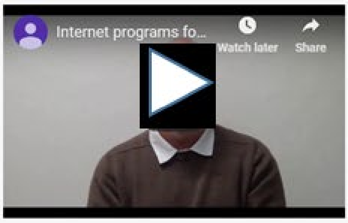

Testimonials x 2

Millie: *"Practising some of the activities can be really difficult at first, and some days you just won't want to do any of it – but it is worth keeping going, because it can actually make a difference to how you feel"*

John: *"With e-mental health services you can track your progress, and even do it in your pyjamas late at night"*

Key motivating quotes (x2) for adherence and ease of use

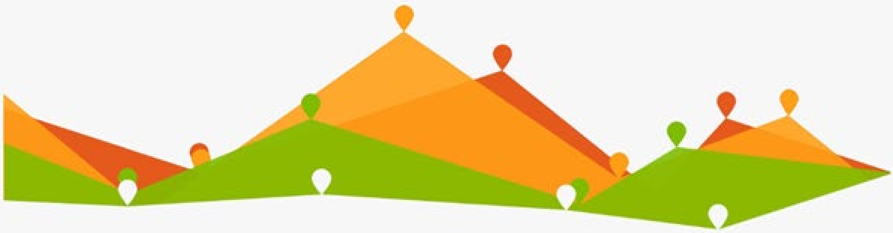

Back Next

Black Dog Institute

*note – underlined text will link out to evidence.
